# Comparison of 3-Year Outcomes between Early and Delayed Invasive Strategies in Older and Younger Adults with Non-ST-Segment Elevation Myocardial Infarction Undergoing New-Generation Drug-Eluting Stent Implantation

**DOI:** 10.3390/jcm11164780

**Published:** 2022-08-16

**Authors:** Yong Hoon Kim, Ae-Young Her, Seung-Woon Rha, Cheol Ung Choi, Byoung Geol Choi, Ji Bak Kim, Soohyung Park, Dong Oh Kang, Ji Young Park, Sang-Ho Park, Myung Ho Jeong

**Affiliations:** 1 Division of Cardiology, Department of Internal Medicine, Kangwon National University School of Medicine, Chuncheon 24289, Korea; 2 Cardiovascular Center, Korea University Guro Hospital, Seoul 08308, Korea; 3 Cardiovascular Research Institute, Korea University College of Medicine, Seoul 02841, Korea; 4 Division of Cardiology, Department of Internal Medicine, Cardiovascular Center, Nowon Eulji Medical Center, Eulji University, Seoul 01830, Korea; 5 Cardiology Department, Soonchunhyang University Cheonan Hospital, Cheonan 31151, Korea; 6 Department of Cardiology, Cardiovascular Center, Chonnam National University Hospital, Gwangju 61469, Korea

**Keywords:** drug-eluting stent, elderly, non-ST-segment elevation myocardial infarction, percutaneous coronary intervention

## Abstract

We evaluated the 3-year clinical outcomes of early invasive (EI) and delayed invasive (DI) strategies in older and younger adults with non-ST-segment elevation myocardial infarction (NSTEMI) undergoing successful new-generation drug-eluting stent (DES) implantation to reflect current real-world practice. Overall, 4513 patients with NSTEMI were recruited from the Korea Acute Myocardial Infarction Registry-National Institute of Health and divided into two groups according to age: group A (age ≥ 65 years, *n* = 2253) and group B (age < 65 years, *n* = 2260). These two groups were further divided into two subgroups: group EI (A1 and B1) and DI (A2 and B2). The primary clinical outcome was the occurrence of major adverse cardiac and cerebrovascular events (MACCEs), defined as all-cause death, recurrent MI (re-MI), any repeat coronary revascularization, or stroke. The secondary clinical outcome was definite or probable stent thrombosis (ST). In both groups A and B, after multivariable-adjusted and propensity score-adjusted analyses, MACCE (group A, *p* = 0.137 and *p* = 0.255, respectively; group B, *p* = 0.171 and *p* = 0.135, respectively), all-cause death, cardiac death (CD), non-CD, re-MI, any repeat revascularization, stroke, and ST rates were similar between the EI and DI groups. When including only those with complex lesions, the primary and secondary clinical outcomes were not significantly different between the EI and DI groups. In the era of new-generation DESs, major clinical outcomes were not significantly different between the EI and DI strategies in both older and younger adults with NSTEMI.

## 1. Introduction

In patients presenting with non-ST-segment elevation (NSTE) myocardial infarction (MI), the results of the Timing of Intervention in Acute Coronary Syndrome (TIMACS) trial [1] demonstrated that the outcomes of individuals who underwent routine early invasive (EI) strategy (coronary angiography [CAG] within 24 h of admission) did not differ greatly from those of individuals who underwent delayed invasive (DI) strategy in preventing the primary outcome, but it could reduce the rate of the composite secondary outcome of death, myocardial infarction, or refractory ischemia and was superior to DI in high-risk patients (*p* = 0.003) during a 6-month follow-up period. In a meta-analysis, the routine invasive strategy significantly reduced 5-year rates of cardiovascular death or MI compared to the selective invasive strategy (*p* = 0.002) [2]. Recently, in the Very Early Versus Deferred Invasive Evaluation Using Computerized Tomography study (VERDICT) [3] with a mean follow-up of 4.3 years, a very early strategy (median time from diagnosis to revascularization = 4.7 h) improved the primary outcome compared with the standard invasive treatment (hazard ratio [HR], 0.81; 95% confidence interval [CI], 0.67–1.01) in the high-risk subgroup, but it did not improve overall long-term clinical outcomes compared with an invasive strategy conducted within 2–3 days in patients with NSTE-acute coronary syndrome (ACS). Hence, pooled analyses of randomized trials [1] or meta-analyses [2,4,5] showed early benefit of the routine intervention, but long-term results are inconsistent, and the optimal timing of percutaneous coronary intervention (PCI) in NSTEMI has yet to be determined. According to the most recent European guidelines [6], the EI strategy is recommended in patients with at least one high-risk criterion, and the recommended diagnostic and interventional strategies for older and younger patients are the same (class I and level of evidence B). The American College of Cardiology/American Heart Association guidelines [7] recommend an EI strategy for initially stabilized high-risk patients with NSTE-ACS and a DI strategy as reasonable for high/intermediate-risk patients (class IIa and level of evidence B). Although information concerning the preferred treatment option between the EI and DI strategies in older and younger patients with NSTEMI could be important for the interventional cardiologist, the available data on this subject are limited. Furthermore, previous studies on the comparative outcomes between the EI and DI strategies were not limited to patients who received new-generation drug-eluting stents (DESs), thereby limiting their findings in reflecting the current real-world practices. In this study, we compared the 3-year major clinical outcomes in older and younger adults with NSTEMI who underwent new-generation DES implantation.

## 2. Methods

### 2.1. Study Population

**Figure 1:**
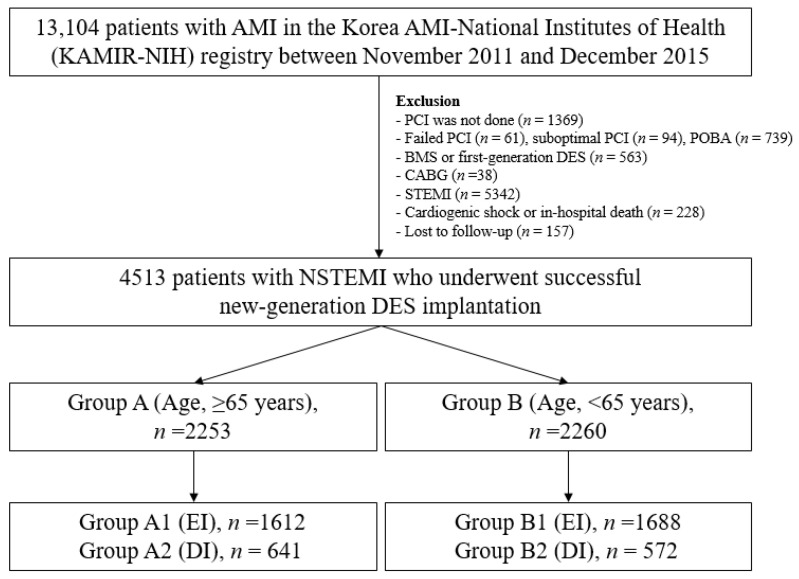
Flowchart. PCI, percutaneous coronary intervention; POBA, plain old balloon angioplasty; BMS, bare-metal stent; DES, drug-eluting stent; CABG, coronary artery bypass graft; STEMI, ST-segment-elevation myocardial infarction; NSTEMI, non-STEMI; EI, early invasive; DI, delayed invasive.

A total of 13,104 patients with acute MI between November 2011 and December 2015 were recruited from the Korea Acute MI Registry-National Institute of Health (KAMIR-NIH) [8]. KAMIR-NIH is a nationwide prospective multicenter registry integrated from 20 high-volume centers in the Republic of Korea. Detailed information on this registry can be found on the website (http://www.kamir.or.kr, accessed on 1 November 2011). All patients aged ≥18 years at the time of hospital admission were included. The following patients were excluded from the study: (1) patients who did not undergo PCI (*n* = 1369, 10.4%); (2) those who underwent unsuccessful PCI (failed PCI (*n* = 61, 0.5%) and suboptimal PCI (*n* = 94, 0.7%)); (3) those who underwent balloon angioplasty (*n* = 739, 5.6%); (4) those who were treated with bare-metal stents or first-generation DESs (*n* = 563, 4.3%); (5) those who underwent coronary artery bypass grafting (*n* = 38, 0.3%); (6) those with ST-segment elevation myocardial infarction (STEMI, *n* = 5342, 40.8%), cardiogenic shock, or in-hospital death (*n* = 228, 1.7%); and (7) those who were unavailable for follow-up (*n* = 157, 1.2%). Overall, 4513 patients with NSTEMI who underwent successful new-generation DES implantation were included (Figure 1). The types of new-generation DESs used are listed in Table 1. These patients were divided into two groups according to their age: group A (age ≥ 65 years, *n* = 2253, 49.9%) and group B (age < 65 years, *n* = 2260, 50.1%). Subsequently, these two groups of patients were further divided into two subgroups: group EI (group A1 (*n* = 1612, 71.5%) or B1 (*n* = 1688, 74.7%)) and DI (group A2 (*n* = 641, 28.5%) and B2 (*n* = 572, 25.3%)) (Figure 1). Trained research coordinators at each center collected patient data using a web-based report form on the Internet-based Clinical Research and Trial management system, supported by a grant (2016-ER6304-02) from the Korean Centers for Disease Control and Prevention since November 2011 (Internet-based Clinical Research and Trial management system study No. C110016). The study was conducted in accordance with the ethical guidelines of the 2004 Declaration of Helsinki. The study was approved by the ethics committee of each participating center and the Chonnam National University Hospital Institutional Review Board ethics committee (CNUH-2011-172). All patients included in the study provided written informed consent prior to enrollment. They also completed a 3-year clinical follow-up via face-to-face interviews, phone calls, or chart reviews. All clinical events were evaluated by an independent event adjudication committee. The event adjudication process has previously been described by KAMIR investigators [8].

### 2.2. Percutaneous Coronary Intervention and Medical Treatment

A transfemoral or transradial approach was performed in accordance with the general guidelines [9]. Aspirin (200–300 mg) and clopidogrel (300–600 mg), ticagrelor (180 mg), or prasugrel (60 mg) were prescribed to the patients as loading doses before PCI. After PCI, all patients were prescribed aspirin (100 mg/day) along with clopidogrel (75 mg/day), ticagrelor (90 mg twice a day), or prasugrel (5–10 mg/day) for at least 1 year. The access site, revascularization strategy, and selection of the DES were left to the discretion of the individual surgeons.

### 2.3. Study Definitions and Clinical Outcomes

NSTEMI was defined as the absence of persistent ST-segment elevation with increased levels of cardiac biomarkers and appropriate clinical context [6,7]. A successful PCI was defined as residual stenosis of <30% and thrombolysis in MI (TIMI) flow grade 3 in the infarct-related artery. EI strategy was defined as CAG performed within 24 h of admission [1]. Glomerular function for estimated glomerular filtration rate (eGFR) was calculated using the Chronic Kidney Disease Epidemiology Collaboration equation [10]. The GRACE risk score [11] was calculated for all patients. Complex lesions were defined as PCI for unprotected left main coronary disease, multivessel PCI, multiple stent implantation (≥3 stents per patient), and cases with a total length of deployed stent >38 mm [12,13]. The primary clinical outcome was the occurrence of major adverse cardiac and cerebrovascular events (MACCE), which was defined by all-cause death, recurrent MI (re-MI), and any repeat coronary revascularization, including target lesion revascularization, target vessel revascularization (TVR), non-TVR, and stroke. According to the American Heart Association/American Stroke Association guidelines, an acute cerebrovascular event resulting in death or neurological deficit for >24 h or the presence of acute infarction demonstrated by imaging studies was defined as stroke [14]. All-cause death was considered a cardiac death (CD) unless an undisputed non-cardiac cause was present [15]. The secondary clinical outcome was definite or probable stent thrombosis (ST) during a 3-year follow-up period. ST was defined according to the definition provided by the Academic Research Consortium [16]. The definitions of re-MI, TLR, TVR, and non-TVR have been published previously [17].

### 2.4. Statistical Analyses

For continuous variables, differences between the groups were evaluated using unpaired *t*-tests. Data are expressed as the mean ± standard deviation or median (interquartile range). For discrete variables, differences between the groups were expressed as counts and percentages and were analyzed using the chi-squared or Fisher’s exact tests. Univariate analysis was performed for all variables in the EI and DI groups with the *p*-value set at <0.05. Subsequently, we performed a multicollinearity test [18] between the included variables to confirm non-collinearity between them (Supplementary Table S1). Variance inflation factor (VIF) values were calculated to measure the degree of multicollinearity among the variables. A VIF of >5 indicated a high correlation [19]. When the tolerance value was <0.1 [20] or the condition index was >10 [19], the presence of multicollinearity was considered. Variables included in the multivariable Cox regression analysis were male sex, left ventricular ejection fraction (LVEF), body mass index, systolic blood pressure, diastolic blood pressure, symptom-to-door time, Killip class 3, hypertension, diabetes mellitus, dyslipidemia, previous PCI, previous heart failure, previous stroke, current smoker, peak creatine kinase myocardial band (CK-MB), peak troponin-I, serum creatinine, eGFR <60 mL/min/1.73 m^2^, high-density lipoprotein cholesterol, low-density lipoprotein cholesterol, GRACE risk score >140, and clopidogrel, ticagrelor, prasugrel, angiotensin-converting enzyme inhibitor or angiotensin receptor blocker, and statin use. Moreover, to adjust for potential confounders, a propensity score (PS)-adjusted analysis was performed using a logistic regression model. We tested all potentially relevant variables, such as baseline clinical, angiographic, and procedural factors (Table 1). The c-statistic for the PS-matched (PSM) analysis in this study was 0.684. Patients in the EI group were matched to those in the DI group (1:1) using the nearest available pair-matching method according to PS. The subjects were matched with a caliper width of 0.01. This procedure yielded 2318 well-matched pairs (Supplementary Table S2). Various clinical outcomes were estimated using a Kaplan–Meier curve analysis, and group differences were compared using the log-rank test. Statistical significance was defined as a two-tailed *p*-value of <0.05. All statistical analyses were performed using SPSS software v. 20 (IBM; Armonk, NY, USA).

## 3. Results

### 3.1. Baseline Characteristics

Table 1 and Supplementary Tables S2 and S3 show the baseline, laboratory, angiographic, and procedural characteristics of the study population. In both groups, A and B, the mean values of peak CK-MB and troponin-I and the number of patients with pre-PCI TIMI flow grade 0/1 were higher in the EI group (group A1 or B1) than in the DI group (group A2 or B2). In contrast, patients who had Killip class 3 had reduced renal function (eGFR, <60 mL/min/1.73 m^2^) and received clopidogrel as discharge medication; the mean serum creatinine level was 1.26 ± 1.34 vs. 1.12 ± 1.15 mg/L in group A2 vs. group A1 (*p* = 0.023), and 1.21 ± 1.73 vs. 1.04 ± 1.27 mg/L in group B2 vs. group B1 (*p* = 0.034); the use of intravascular ultrasound/optical coherent tomography/fractional flow rate was higher in the DI group than in the EI group. In group A, the mean value of LVEF, number of current smokers, and prescription rates of ticagrelor, ACEIs, or ARBs as discharge medications were higher in the EI group (group A1) than in the DI group (group A2). However, the mean age of enrolled patients; mean values of BMI, SBP, and DBP; number of patients with dyslipidemia and multivessel disease; and mean number of deployed stents were higher in the DI group (group A2) than in the EI group (group A1). In group B, the prescription rates of prasugrel, beta-blockers, and statin; use of glycoprotein IIb/IIIa inhibitors; and transradial approach rate were higher in the EI group (group B1) than in the DI group (group B2). In contrast, the number of patients with previous MI and PCI and higher GRACE risk scores (>140) were higher in the DI group (group B2) than in the EI group (group B1) (Table 1).

### 3.2. Clinical Outcomes

**Figure 2:**
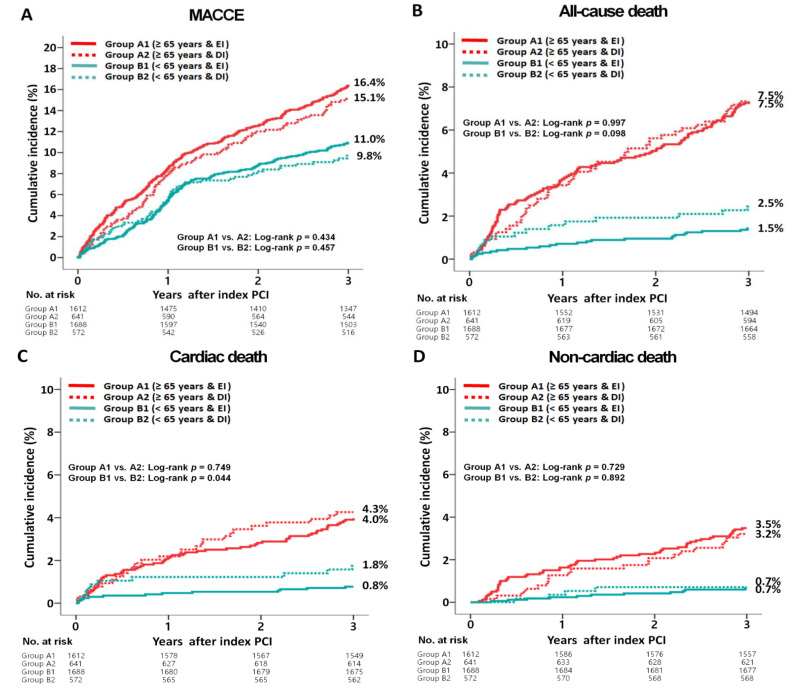

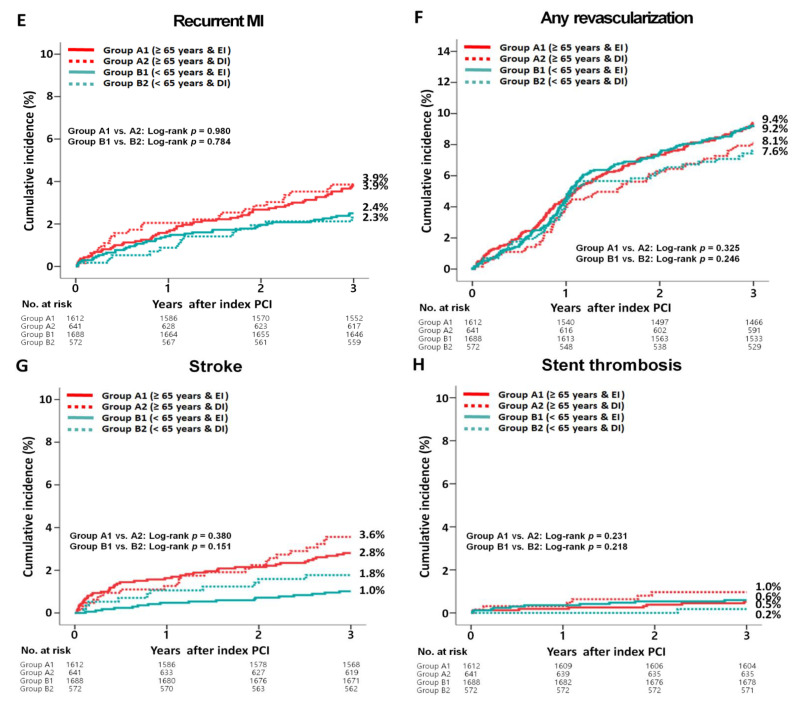
Kaplan-Meier curved analysis for MACCE (**A**), all-cause death (**B**), cardiac death (**C**), non-cardiac death (**D**), recurrent MI (**E**), any repeat revascularization (**F**), stroke (**G**), and stent thrombosis (**H**). MACCE, major adverse cardiac and cerebrovascular events; MI, myocardial infarction; PCI, percutaneous coronary intervention; EI, early invasive; DI, delayed invasive.

The 3-year major clinical outcomes are summarized in Table 2 and Figure 2. After multivariable-adjusted analysis, in group A, the MACCE (Figure 2A, adjusted HR (aHR), 1.198; 95% CI, 0.944–1.521; *p* = 0.137), all-cause death (Figure 2B, aHR, 1.150; *p* = 0.434), CD (Figure 2C, aHR, 1.100; *p* = 0.692), non-CD (Figure 2D, aHR, 1.207; *p* = 0.485), re-MI (Figure 2E, aHR, 1.061; *p* = 0.809), any repeat revascularization (Figure 2F, aHR, 1.247; *p* = 0.186), stroke (Figure 2G, aHR, 1.255; *p* = 0.394), and ST (definite or probable, Figure 2H, aHR, 2.969; 95% CI, 0.978–9.017; *p* = 0.055) rates were not significantly different between groups A1 and A2. In group B, the MACCE (aHR, 1.236; 95% CI, 0.913–1.673; *p* = 0.171), all-cause death (aHR, 1.065; *p* = 0.869), CD (aHR, 1.359; *p* = 0.527), non-CD (aHR, 1.447; *p* = 0.570), re-MI (aHR, 1.259; *p* = 0.478), any repeat revascularization (aHR, 1.289; *p* = 0.145), stroke (aHR, 1.523; *p* = 0.299), and ST (definite or probable, aHR, 4.152; 95% CI, 0.501–32.82; *p* = 0.101) rates were not significantly different between groups B1 and B2. In the total study population, MACCE (aHR, 1.199; 95% CI, 0.995–1.445; *p* = 0.056), all-cause death (aHR, 1.078; *p* = 0.636), CD (aHR, 1.060; *p* = 0.780), non-CD (aHR, 1.281; *p* = 0.313), re-MI (aHR, 1.034; *p* = 0.864), any repeat revascularization (aHR, 1.258; *p* = 0.056), stroke (aHR, 1.351; *p* = 0.175), and ST (definite or probable, aHR, 1.091; 95% CI, 0.449–2.651; *p* = 0.847) rates were not significantly different between the EI (group A1+B1) and DI (group A2+B2) groups (Table 2). These results were confirmed after PS-adjusted analysis. After PS-adjusted analysis in both groups A and B, the primary and secondary clinical outcomes were not significantly different between groups A1 and A2 or groups B1 and B2 (Table 2). To provide more meaningful insights with a cut-off age of 75 or 80 years, the major clinical outcomes were reanalyzed according to the two cut-off ages of the study population (Supplementary Tables S4 and S5). It was observed that regardless of the cut-off age, the primary and secondary clinical outcomes were not significantly different between groups A1 and A2 or groups B1 and B2.

**Figure 3:**
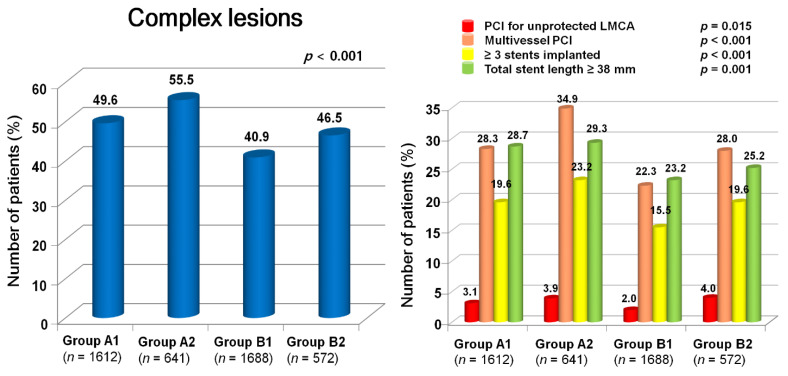
Distribution of complex lesions in the 4 groups. Group A1, ≥65 years and early invasive; Group A2, ≥65 years and delayed invasive; Group B1, <65 years and early invasive; Group B2, <65 years and delayed invasive, PCI percutaneous coronary intervention, LMCA left main coronary artery.

**Figure 4:**
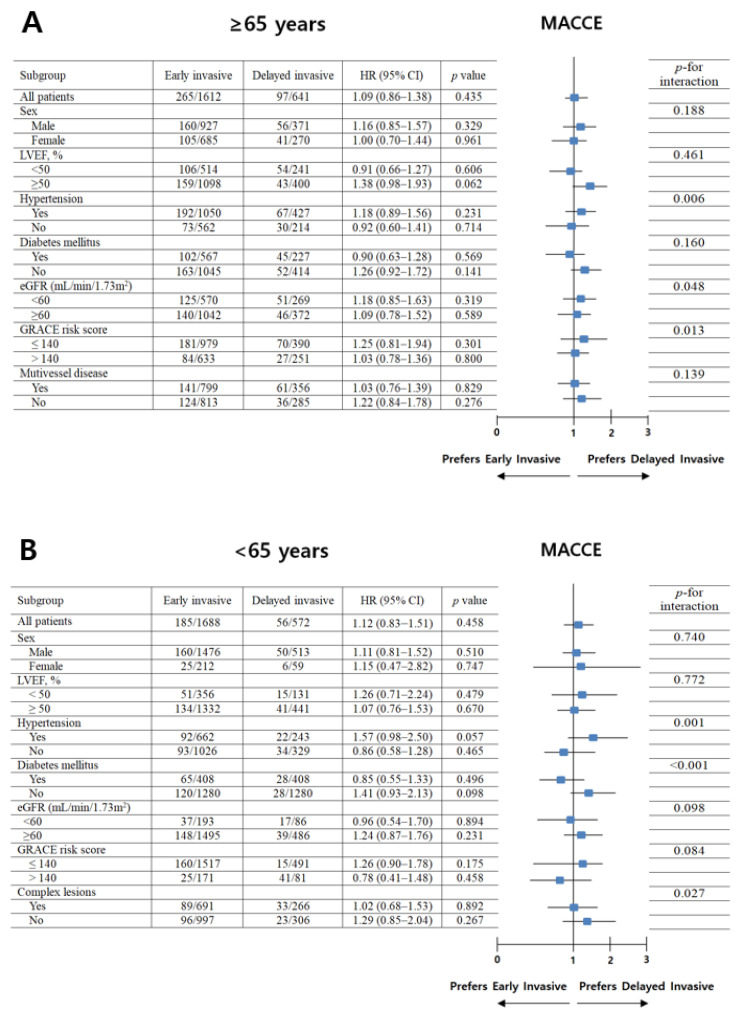
Subgroup analysis for MACCE in older (**A**) and younger (**B**) adults. MACCE, major adverse cardiac events; HR, hazard ratio; CI, confidence interval; LVEF, left ventricular ejection fraction; eGFR, estimated glomerular filtration rate; GRACE, Global Registry of Acute Coronary Events.

For further assessment of major clinical outcomes between the EI and DI groups of groups A and B, we compared these major clinical outcomes by limiting the study population to patients with complex lesions (Table 3). The number of patients with complex lesions in each group was >40% (group A1, 49.6%; group A2, 55.5%; group B1, 40.9%; group B2, 46.5%) (Figure 3). The MACCE rates were similar between the EI and DI groups (group A1 vs. group A2; aHR, 1.149; 95% CI, 0.843–1.564; *p* = 0.379; group B1 vs. group B2; aHR, 1.136; 95% CI, 0.754–1.713; *p* = 0.542) (Table 3). The ST (definite or probable) rates were also similar between the EI and DI groups (group A1 vs. group A2; aHR, 3.777; 95% CI, 0.673–116.94; *p* = 0.139; group B1 vs. group B2; aHR, 1.140; 95% CI, 0.030–43.82; *p* = 0.944, Table 3). Additionally, all-cause death, CD, non-CD, re-MI, any repeat revascularization, and stroke rates were not significantly different between the EI and DI groups after adjustment (Table 3). Figure 4 shows the subgroup analysis for MACCE in groups A and B. The results of the subgroup analysis using the Cox logistic regression model revealed that all subgroups, except for those showing significant *p*-for-interaction, demonstrated comparable MACCE rates in this study.

## 4. Discussion

The main findings of this prospective observational study were as follows: (1) in both older and younger groups, after multivariable-adjusted and PS-adjusted analyses, MACCE, all-cause death, CD, non-CD, re-MI, any repeat revascularization, stroke, and ST (definite or probable) rates were similar between the EI and DI groups; (2) even after limiting the study population to patients who had complex lesions in both older and younger groups, the primary and secondary clinical outcomes were not significantly different between the EI and DI groups.

The merits of the EI strategy include early identification of significant lesions, early revascularization, and facilitation of earlier discharge from a facility [21]. In contrast, the DI strategy may provide adequate time for optimal medical treatment to decrease the thrombus burden and improve plaque stability [21]. In general, older individuals presenting with ACS tend to have clinical complexity, frailty, and high-risk coronary lesions [22]. Moreover, the clinical presentation of NSTE-ACS in older adults is atypical [10], and the electrocardiographic changes are less frequent in older patients than in younger patients [23]. Because of evidence-based therapy, there was a significant decrease in mortality and morbidities associated with ACS [24]. However, the improvements in ACS treatment strategy have not equally improved outcomes for older adults [7]. Additionally, there is a paucity of evidence to guide the selection of the EI or DI strategy in elderly patients with NSTE-ACS [25]. Although previous reports [26,27] demonstrated significant beneficial effects of the EI strategy compared with conservative treatment in elderly patients with NSTE-ACS, these studies were not performed in the era of new-generation DESs and did not compare clinical outcomes between the EI and DI strategies. We know that the 3-year follow-up period in this study was insufficient to estimate long-term clinical outcomes. To overcome insufficient information concerning comparative clinical outcomes between the EI and DI strategies in older and younger adults with NSTEMI undergoing successful new-generation DES implantation, we attempted to investigate the 3-year clinical outcomes, which were not a long time. The definition of older adults is controversial. In general, a person aged ≥60 or 65 years is considered an older adult [28]. The average age at which individuals experience a first heart attack is 65.8 years for men and 70.4 years for women [29]. Additionally, based on the Consensus Development Conference on Diabetes and Older Adults (age ≥65 years) convened by the American Diabetes Association in February 2012 [30] and another report [31], which showed that multimorbidity and polypharmacy are highly prevalent among adults aged ≥65 years, we set the cut-off age at ≥65 years for older adults in our study.

In the case of neointimal hyperplasia and repeated revascularization, a DES, in which a pharmaceutically active agent is coated onto a bare-metal stent (BMS) along with a drug-carrying polymer, is used to lower the risks posed by BMSs [7]. Although DESs are carefully designed to reduce ST, the risk of late ST and restenosis is seen with DES use in clinical trials [7,32]. The introduction of the 1G-DES (Cypher and Taxus) revolutionized the field of interventional cardiology, but second-generation DESs (2G-DES; Xience, Promus) are the gold standard of stent technology because they not only resolved the problems associated with 1G-DES (such as inflammation and restenosis) but also decreased the mortality rate [33].

The current guidelines suggest that older patients with NSTE-ACS should be considered for invasive management with CAG and PCI [6,7]. However, the key study underpinning the current guidelines [6,7] was the TIMACS trial [1]. Although this study [1] showed valuable results for understanding the beneficial effect of EI CAG in patients with ACS, this study was conducted between April 2003 and June 2008; approximately 45% of the cases used BMSs, and the type of DES was not confined to the new-generation DES. Additionally, <60% of the patients underwent PCI. In our study, in both older and younger groups, the major clinical outcomes were not significantly different between the EI and DI groups after adjustments (multivariable or PS-adjusted) during a 3-year follow-up period. Regarding the limitations of the TIMACS trial [1], our study results could be more impactful with respect to reflecting the current real-world practices. As shown in Table 3, we performed additional analysis to clearly estimate long-term clinical outcomes between the EI and DI groups. Even after considering patients with complex lesions [16,17], the 3-year major clinical outcomes were not significantly different between the two groups (Table 3). Subgroup analyses for MACCE in groups A and B (Figure 4) showed that all subgroups except for those showing significant *p*-for-interaction had comparable MACCE rates.

The proportion of men decreased with age in group A (≥65 years) compared with group B (<65 years) in our study. Additionally, comorbidities including hypertension, diabetes mellitus, previous MI, previous HF, previous stroke, and renal insufficiency (eGFR <60 mL/min/1.73 m^2^) were more prevalent in group A than in group B (Table 1). Therefore, the patient characteristics in this study are consistent with previously published data [29,34]. This increasing prevalence of cardiovascular disease with aging has been attributed to several age-related changes, including vascular wall elasticity, coagulation, the hemostatic system, and endothelial dysfunction [35,36,37]. Hence, age-related decline in organ function could increase cardiovascular diseases [37].

An age subgroup analysis [31] from the Treat Angina with Aggrastat and Determine Cost of Therapy with an Invasive or Conservative Strategy—Thrombolysis in Myocardial Infarction 18 (TACTICS-TIMI 18) trial [38] showed that the EI strategy yielded a greater absolute (4.1% vs. 1%) and relative (42% vs. 20.4%) risk reduction in mortality or MI at 30 days in the ≥65 years of age subgroup compared with younger patients. However, this benefit coexisted with a 3-fold higher risk of major bleeding with the EI strategy in patients ≥75 years of age (16.6% vs. 6.5%; *p* = 0.009). Thus, compared with younger patients, older patients gain greater absolute and relative benefits from the EI strategy but with increased bleeding risk [10]. However, similar to the TIMACS trial [1], the types of deployed stents were not confined to new-generation DESs in these studies [31,38].

The current guideline [6] suggests that the management of older patients should be based on ischemic and bleeding risks, estimated life expectancy, comorbidities, the need for non-cardiac surgery, quality of life, frailty, cognitive and functional impairment, patient values and preferences, and estimated risks and benefits of revascularization. We agree with this suggestion. Interestingly, in the era of new-generation DESs, the major clinical outcomes were not significantly different between the EI and DI strategies in both older and younger adults with NSTEMI during a 3-year follow-up period in our study. In the present study, although the population size may have been insufficient to provide meaningful results, 20 tertiary high-volume university hospitals participated in the registry. Therefore, we believe that our results could provide helpful information to interventional cardiologists in terms of the long-term effects of the EI and DI strategies in older and younger adults with NSTEMI undergoing successful new-generation DES implantation. Based on our results, we can conclude that elderly patients with several comorbidities and a relatively mild NSTEMI would receive a more “planned” (hence delayed) treatment. It is reassuring to note that this does not lead to inferior clinical outcomes. However, we could not completely explain the comparable clinical outcomes between the various study groups. It may be an important shortcoming of the non-randomized registry study.

In our study, although the number of patients with multivessel disease (average > 55%) and type B2/C lesions (average > 80%) were higher, the LVEF was normal (average > 62%). The number of patients with multivessel disease and type B2/C lesions in our study may have increased after applying the exclusion criteria, as shown in Figure 1. Moreover, the baseline characteristics of our study are similar to those in recent publications based on the KAMIR-NIH [39,40].

This study had some limitations. First, although this study was based on a prospective observational registry, it is not a randomized controlled study, and there may have been selection bias. Second, bleeding is a serious complication that occurs after PCI in older adults [26,27]; however, anti-platelet therapy after 1 year index PCI was different among physicians; therefore, we could not include bleeding as an outcome parameter in our study during the 3-year follow-up period—this is a major shortcoming of our study. Third, because we set the cut-off age for older adults at ≥65 years, our results may change according to different cut-off ages. Fourth, despite the multivariable and PS-adjusted analyses, variables that were not included in the data registry may have affected the study outcome. Fifth, the 3-year follow-up period was insufficient to evaluate long-term adverse events. Sixth, although the number of coronary bifurcation lesions, type and incidence of procedural complications (no-reflow, coronary dissections, etc.), characteristics of calcified coronary lesions, and use of rotational atherectomy may have impacted the outcome and important variables for long-term prognosis, these variables were not mandatory in the KAMIR-NIH data. Hence, we could not provide this information in our study. Finally, there were substantial differences between the EI and DI cohorts. For example, the fact that peak troponin was higher and TIMI flow 0/1 was more often present in the EI groups indicates a selection bias for more severe NSTEMI cases being treated earlier (which is to be expected in the registry setting). Yet, the DI group had more comorbidities. The PS-adjusted analysis attempts to compensate for this but is still not ideal. To really prove that the EI strategy does not improve outcomes compared with the DI strategy, a randomized trial is required.

## 5. Conclusions

In conclusion, in both older and younger adults with NSTEMI, the EI and DI strategies showed comparable clinical outcomes after successful new-generation DES implantation during a 3-year follow-up period. However, to clarify the differences in clinical outcomes between these two reperfusion strategies in those patients, further randomized, large-scale, and long-term follow-up studies are needed.

## Figures and Tables

**Table 1:** Baseline characteristics and discharge medications.

Variables	Group A (Age, ≥65 Years, *n* = 2253)			Group B (Age, <65 Years, *n* = 2260)		
	Group A1 Early Invasive (*n* = 1612)	Group A2 Delayed Invasive (*n* = 641)	*p* Value	Group B1 Early Invasive (*n* = 1688)	Group B2 Delayed Invasive (*n* = 572)	*p* Value
Male, *n* (%)	927 (57.5)	371 (57.9)	0.872	1476 (87.4)	513 (89.7)	0.153
Age, years	74.3 ± 5.8	75.0 ± 5.9	0.007	54.4 ± 7.3	54.5 ± 7.2	0.760
LVEF, %	53.2 ± 10.6	51.6 ± 12.3	0.005	55.9 ± 9.4	55.1 ± 10.9	0.149
BMI, kg/m^2^	23.2 ± 3.1	23.5 ± 3.3	0.048	25.0 ± 3.2	24.8 ± 3.1	0.120
SBP, mmHg	133.5 ± 26.4	135.4 ± 25.8	<0.001	137.0 ± 25.8	139.2 ± 25.8	0.087
DBP, mmHg	80.4 ± 15.7	81.3 ± 14.8	0.038	83.9 ± 15.8	83.8 ± 15.1	0.874
Symptom-to-door time, h	8.0 (3.0–28.6)	8.8 (2.7–45.3)	0.054	5.8 (2.0–19.3)	4.5 (1.6–23.9)	0.181
Door-to-balloon time, h	6.0 (2.9–16.1)	46.4 (31.1–71.6)	<0.001	6.9 (3.0–16.1)	43.2 (29.8–58.6)	<0.001
Killip class 3	181 (11.2)	98 (15.3)	0.011	65 (3.9)	34 (5.9)	0.044
Hypertension, *n* (%)	1050 (65.1)	427 (66.6)	0.505	662 (39.2)	243 (42.5)	0.183
Diabetes mellitus, *n* (%)	567 (35.2)	227 (35.4)	0.914	408 (24.2)	154 (26.9)	0.198
Dyslipidemia, *n* (%)	154 (9.6)	83 (12.9)	0.022	225 (13.3)	92 (16.1)	0.109
Previous MI, *n* (%)	136 (8.4)	48 (7.5)	0.496	73 (4.3)	388 (6.6)	0.033
Previous PCI, *n* (%)	112 (6.9)	33 (5.1)	0.128	66 (3.9)	34 (5.9)	0.046
Previous CABG, *n* (%)	6 (0.4)	3 (0.5)	0.720	2 (0.1)	1 (0.2)	0.749
Previous HF, *n* (%)	27 (1.7)	15 (2.3)	0.302	9 (0.5)	6 (1.0)	0.230
Previous stroke, *n* (%)	124 (7.7)	57 (8.9)	0.346	60 (3.6)	23 (4.0)	0.608
Current smokers, *n* (%)	324 (20.1)	102 (15.9)	0.023	921 (54.6)	309 (54.0)	0.846
Peak CK-MB, mg/dL	20.9 (6.4–78.6)	13.9 (5.0–42.6)	<0.001	29.0 (7.2–99.0)	15.6 (4.6–56.7)	<0.001
Peak Troponin-I, ng/mL	10.6 (2.1–22.1)	4.7 (1.1–18.9)	<0.001	14.3 (2.8–23.1)	5.4 (1.0–21.1)	<0.001
Blood glucose, mg/dL	158.6 ± 72.7	162.1 ± 80.2	0.338	153.6 ± 73.4	158.9 ± 79.6	0.157
Hs-CRP (mg/dL)	1.53 ± 3.24	1.78 ± 7.72	0.440	1.07 ± 2.50	1.11 ± 2.10	0.687
Serum creatinine (mg/L)	1.12 ± 1.15	1.26 ± 1.34	0.023	1.04 ± 1.27	1.21 ± 1.73	0.034
eGFR < 60 mL/min/1.73 m^2^, *n* (%)	570 (35.4)	269 (42.0)	0.003	193 (11.4)	86 (15.0)	0.027
Total cholesterol, mg/dL	171.9 ± 43.3	171.7 ± 44.1	0.900	188.5 ± 43.1	185.3 ± 41.9	0.117
Triglyceride, mg/L	111.7 ± 71.8	112.8 ± 82.7	0.771	152.7 ± 96.3	156.2 ± 94.3	0.523
HDL cholesterol, mg/L	43.1 ± 11.4	44.5 ± 82.7	0.013	42.1 ± 10.8	42.2 ± 10.6	0.913
LDL cholesterol, mg/L	108.7 ± 34.7	106.0 ± 35.3	0.101	120.2 ± 36.8	116.9 ± 35.3	0.053
GRACE risk score	151.2 ± 34.5	154.4 ± 36.7	0.058	105.8 ± 28.4	106.5 ± 32.3	0.676
>140, *n* (%)	979 (60.7)	390 (60.8)	0.961	171 (10.1)	81 (14.2)	0.011
Atrial fibrillation, *n* (%)	93 (5.8)	44 (6.9)	0.329	26 (1.5)	13 (2.3)	0.265
ST-depression, *n* (%)	392 (24.3)	157 (24.5)	0.930	334 (19.8)	103 (18.0)	0.352
T-wave inversion, *n* (%)	370 (23.0)	155 (24.2)	0.534	291 (17.2)	119 (20.8)	0.060
Discharge medications, *n* (%)						
Aspirin, *n* (%)	1600 (99.3)	635 (99.1)	0.645	1678 (99.4)	568 (99.3)	0.778
Clopidogrel, *n* (%)	1251 (77.6)	540 (84.2)	<0.001	1065 (63.1)	406 (71.0)	0.001
Ticagrelor, *n* (%)	283 (17.6)	77 (12.0)	0.001	361 (21.4)	109 (19.1)	0.257
Prasugrel, *n* (%)	78 (4.8)	24 (3.7)	0.106	262 (15.5)	57 (10.0)	0.001
BBs, *n* (%)	1354 (84.0)	542 (84.6)	0.742	1491 (88.3)	485 (84.8)	0.029
ACEIs or ARBs, *n* (%)	1361 (84.4)	506 (78.9)	0.002	1423 (84.3)	462 (80.8)	0.051
Statin, *n* (%)	1534 (95.2)	601 (93.8)	0.178	1631 (96.6)	541 (94.6)	0.033
Anticoagulant, *n* (%)	50 (3.1)	25 (3.9)	0.362	11 (0.7)	10 (1.7)	0.024
Infarct-related artery						
Left main, *n* (%)	50 (3.1)	25 (3.9)	0.362	33 (2.0)	23 (4.0)	0.008
LAD, *n* (%)	684 (42.4)	286 (44.6)	0.346	723 (42.8)	238 (41.6)	0.625
LCx, *n* (%)	400 (24.8)	141 (22.0)	0.172	459 (27.2)	150 (26.2)	0.663
RCA, *n* (%)	478 (29.7)	189 (29.5)	0.959	473 (28.0)	161 (28.1)	0.957
Multivessel disease, *n* (%)	971 (60.2)	423 (66.0)	0.011	811 (48.0)	300 (52.4)	0.073
ACC/AHA type B2/C lesions	1373 (85.2)	544 (84.9)	0.854	1413 (83.7)	467 (81.6)	0.271
Pre-PCI TIMI flow grade 0/1	633 (39.3)	199 (31.0)	<0.001	760 (45.0)	177 (30.9)	<0.001
GP IIb/IIIa inhibitor	133 (8.3)	43 (6.7)	0.258	174 (10.3)	41 (7.2)	0.026
Transradial approach	781 (48.4)	309 (48.2)	0.926	959 (56.8)	292 (51.0)	0.017
IVUS/OCT, *n* (%)	346 (21.5)	174 (27.1)	0.004	421 (24.9)	202 (35.3)	<0.001
FFR, *n* (%)	27 (1.7)	23 (3.6)	0.010	33 (2.0)	24 (4.2)	0.005
Drug-eluting stents ^a^						
ZES, *n* (%)	374 (23.2)	155 (24.2)	0.621	419 (24.8)	142 (24.8)	0.999
EES, *n* (%)	860 (53.3)	332 (51.8)	0.504	878 (52.0)	294 (51.4)	0.809
BES, *n* (%)	326 (20.2)	144 (22.5)	0.237	340 (20.1)	125 (21.9)	0.402
Others, *n* (%)	52 (3.2)	10 (1.6)	0.032	51 (3.0)	11 (1.9)	0.184
Stent diameter (mm)	3.04 ± 0.40	3.03 ± 0.41	0.531	3.12 ± 0.43	3.10 ± 0.44	0.196
Stent length (mm)	30.2 ± 14.4	31.1 ± 14.9	0.205	28.6 ± 13.2	29.8 ± 14.5	0.074
Number of stents	1.22 ± 0.46	1.26 ± 0.50	0.044	1.17 ± 0.42	1.22 ± 0.47	0.030

Values are means ± standard deviation or median (interquartile range) or numbers and percentages. The *p* values for continuous data were obtained from the unpaired t-test. The *p* values for categorical data from chi-square or Fisher’s exact test. LVEF, left ventricular ejection fraction; BMI, body mass index; SBP, systolic blood pressure; DBP, diastolic blood pressure; MI, myocardial infarction; PCI, percutaneous coronary intervention; CABG, coronary artery bypass graft; HF, heart failure; CK-MB, creatine kinase myocardial band; Hs-CRP, high sensitivity C-reactive protein; eGFR, estimated glomerular filtration rate; HDL, high-density lipoprotein; LDL, low-density lipoprotein; GRACE, Global Registry of Acute Coronary Events; BBs, ß-blockers; ACEIs, angiotensin-converting enzyme inhibitors; ARBs, angiotensin receptor blockers; LAD, left anterior descending artery; LCx, left circumflex artery; RCA, right coronary artery; ACC/AHA, American College of Cardiology/American Heart Association; TIMI, thrombolysis in myocardial infarction; GP, glycoprotein; IVUS, intravascular ultrasound; OCT, optical coherence tomography; FFR, fractional flow reserve; ZES, zotarolimus-eluting stent; EES, everolimus-eluting stent; BES, biolimus-eluting stent. ^a^ Drug-eluting stents were composed of ZES (Resolute Integrity stent; Medtronic, Inc., Minneapolis, MN), EES (Xience Prime stent, Abbott Vascular, Santa Clara, CA; or Promus Element stent, Boston Scientific, Natick, MA), and BES (BioMatrix Flex stent, Biosensors International, Morges, Switzerland; or Nobori stent, Terumo Corporation, Tokyo, Japan).

**Table 2:** Comparison of clinical outcomes at 2 years.

	**Group A (Age, ≥65 Years, *n* = 2253)**								
**Outcomes**	**Group A1** **Early Invasive** **(*n* = 1612)**	**Group A2** **Delayed Invasive** **(*n* = 641)**	**Log–Rank**	**Unadjusted**		**Multivariable-Adjusted ^a^**		**Propensity score-Adjusted**	
				**HR (95% CI)**	***p***	**HR (95% CI)**	***p***	**HR (95% CI)**	***p***
MACCE	265 (16.4)	97 (15.1)	0.434	1.097 (0.869–1.384)	0.435	1.198 (0.944–1.521)	0.137	1.176 (0.889–1.500)	0.255
All-cause death	118 (7.5)	47 (7.5)	0.997	0.999 (0.713–1.401)	0.997	1.150 (0.810–1.633)	0.434	1.269 (0.850–1.894)	0.244
Cardiac death	63 (4.0)	27 (4.3)	0.749	0.929 (0.592–1.458)	0.749	1.100 (0.687–1.761)	0.692	1.127 (0.694–1.913)	0.659
Non-cardiac death	55 (3.5)	20 (3.2)	0.729	1.095 (0.656–1.826)	0.729	1.207 (0.712–2.043)	0.485	1.487 (0.803–2.753)	0.207
Recurrent MI	60 (3.9)	24 (3.9)	0.980	0.994 (0.619–1.595)	0.980	1.061 (0.654–1.722)	0.809	1.035 (0.584–1.653)	0.907
Any repeat revascularization	146 (9.4)	50 (8.1)	0.325	1.175 (0.852–1.620)	0.326	1.247 (0.899–1.730)	0.186	1.236 (0.843–1.710)	0.277
Stroke	44 (2.8)	22 (3.6)	0.380	0.796 (0.477–1.327)	0.381	1.255 (0.745–2.114)	0.394	1.067 (0.570–2.000)	0.839
ST (definite or probable)	8 (0.5)	6 (1.0)	0.231	0.529 (0.184–1.525)	0.239	2.969 (0.978–9.017)	0.055	1.490 (0.421–5.281)	0.537
	**Group B (Age, <65 Years, *n* = 2260)**								
**Outcomes**	**Group B1** **Early Invasive** **(*n* = 1688)**	**Group B2** **Delayed Invasive (*n* = 572)**	**Log–Rank**	**Unadjusted**		**Multivariable–Adjusted ^a^**		**Propensity score–Adjusted**	
				**HR (95% CI)**	***p***	**HR (95% CI)**	***p***	**HR (95% CI)**	***p***
MACCE	185 (11.0)	56 (9.8)	0.457	1.120 (0.831–1.510)	0.458	1.236 (0.913–1.673)	0.171	1.317 (0.918–1.890)	0.135
All-cause death	24 (1.5)	14 (2.5)	0.098	0.577 (0.299–1.116)	0.102	1.065 (0.506–2.239)	0.869	1.583 (0.614–4.085)	0.342
Cardiac death	13 (0.8)	10 (1.8)	0.044	0.438 (0.192–0.999)	0.050	1.359 (0.525–3.517)	0.527	1.024 (0.212–2.984)	0.925
Non-cardiac death	11 (0.7)	4 (0.7)	0.892	0.924 (0.294–2.901)	0.892	1.447 (0.405–5.172)	0.570	1.505 (0.517–6.102)	0.342
Recurrent MI	42 (2.4)	13 (2.3)	0.784	1.091 (0.586–2.032)	0.784	1.259 (0.666–2.382)	0.478	1.147 (0.746–2.411)	0.717
Any repeat revascularization	155 (9.2)	43 (7.6)	0.246	1.221 (0.871–1.711)	0.247	1.289 (0.917–1.813)	0.145	1.347 (0.921–2.018)	0.149
Stroke	17 (1.0)	10 (1.8)	0.151	0.569 (0.260–1.242)	0.157	1.523 (0.688–3.369)	0.299	1.446 (0.551–3.109)	0.454
ST (definite or probable)	10 (0.6)	1 (0.2)	0.218	3.376 (0.432–26.37)	0.246	4.152 (0.501–32.82)	0.101	2.984 (0.310–23.68)	0.344
	**Early Invasive**	**Delayed Invasive**							
**Outcomes**	**Group A1 + B1** **(*n* = 3300)**	**Group A2 + B2** **(*n* = 1213)**	**Log–Rank**	**Unadjusted**		**Multivariable–Adjusted ^a^**		**Propensity Score–Adjusted**	
				**HR (95% CI)**	***p***	**HR (95% CI)**	***p***	**HR (95% CI)**	***p***
MACCE	450 (13.6)	153 (12.6)	0.380	1.086 (0.904–1.304)	0.380	1.199 (0.995–1.445)	0.056	1.225 (0.998–1.528)	0.071
All-cause death	142 (4.3)	61 (5.1)	0.295	0.852 (0.631–1.150)	0.295	1.078 (0.790–1.470)	0.636	1.130 (0.798–1.630)	0.512
Cardiac death	76 (2.3)	37 (3.1)	0.154	0.752 (0.508–1.144)	0.155	1.060 (0.704–1.595)	0.780	1.058 (0.655–1.521)	0.807
Non-cardiac death	66 (2.0)	24 (2.0)	0.980	1.006 (0.631–1.605)	0.980	1.281 (0.792–2.074)	0.313	1.451 (0.821–2.566)	0.200
Recurrent MI	102 (3.2)	37 (3.1)	0.960	1.010 (0.693–1.471)	0.960	1.034 (0.706–1.516)	0.864	1.029 (0.654–1.498)	0.902
Any repeat revascularization	301 (9.3)	93 (7.9)	0.132	1.195 (0.947–1.508)	0.133	1.258 (0.994–1.591)	0.056	1.235 (0.975–1.575)	0.075
Stroke	61 (1.9)	32 (2.7)	0.095	0.696 (0.454–1.067)	0.097	1.351 (0.875–2.087)	0.175	1.037 (0.635–1.812)	0.792
ST (definite or probable)	18 (0.6)	7 (0.6)	0.893	0.942 (0.393–2.255)	0.893	1.091 (0.449–2.651)	0.847	1.001 (0.351–2.553)	0.999

MACCE, major adverse cardiac and cerebrovascular events; ST, stent thrombosis; HR, hazard ratio; CI, confidence interval; LVEF, left ventricular ejection fraction; BMI, body mass index; SBP, systolic blood pressure; DBP, diastolic blood pressure; DM, diabetes mellitus; PCI, percutaneous coronary intervention; HF, heart failure; CK-MB, creatine kinase myocardial band; eGFR, estimated glomerular filtration rate; HDL, high-density lipoprotein; LDL, low-density lipoprotein; GRACE, Global Registry of Acute Coronary Events; ACEIs, angiotensin-converting enzyme inhibitors; ARBs, angiotensin receptor blockers. ^a^ Adjusted by male sex, LVEF, BMI, SBP, DBP, symptom-to-door time, Killip class 3, hypertension, DM, dyslipidemia, previous PCI, previous HF, previous stroke, current smoker, peak CK-MB, peak troponin-I, serum creatinine, eGFR < 60 mL/min/1.73 m^2^, HDL-cholesterol, LDL-cholesterol, GRACE risk score >140, clopidogrel, ticagrelor, prasugrel, ACEI or ARB, and statin.

**Table 3:** Comparison of clinical outcomes in patient with complex coronary lesions.

	**Group A (Age, ≥65 Years, *n* = 2253)**						
**Outcomes**	**Group A1** **Early Invasive** **(*n* = 799)**	**Group A2** **Delayed Invasive** **(*n* = 356)**	**Log-Rank**	**Unadjusted**		**Multivariable-Adjusted ^a^**	
				**HR (95% CI)**	***p***	**HR (95% CI)**	***p***
MACCE	141 (17.6)	61 (17.1)	0.829	1.034 (0.765–1.396)	0.829	1.149 (0.843–1.564)	0.379
All-cause death	64 (8.2)	27 (7.7)	0.814	1.056 (0.673–1.655)	0.814	1.254 (0.784–2.006)	0.345
Cardiac death	31 (4.0)	16 (4.5)	0.632	0.863 (0.472–1.578)	0.632	1.021 (0.539–1.934)	0.949
Non-cardiac death	33 (4.2)	11 (3.2)	0.404	1.336 (0.675–2.643)	0.406	1.616 (0.794–3.286)	0.185
Recurrent MI	31 (4.0)	14 (4.1)	0.966	0.986 (0.525–1.854)	0.966	1.097 (0.574–2.097)	0.780
Any repeat revascularization	76 (9.9)	35 (10.3)	0.893	0.973 (0.652–1.452)	0.893	1.041 (0.691–1.568)	0.849
Stroke	25 (3.2)	14 (4.1)	0.490	0.795 (0.413–1.529)	0.491	1.338 (0.688–2.601)	0.391
ST (definite or probable)	4 (0.5)	3 (0.9)	0.488	0.592 (0.133–2.646)	0.493	3.777 (0.673–16.94)	0.139
	**Group B (Age, <65 Years, *n* = 977)**						
**Outcomes**	**Group B1** **Early Invasive** **(*n* = 691)**	**Group B2** **Delayed Invasive** **(*n* = 286)**	**Log-Rank**	**Unadjusted**		**Multivariable-Adjusted ^a^**	
				**HR (95% CI)**	***p***	**HR (95% CI)**	***p***
MACCE	89 (12.9)	33 (12.4)	0.892	1.028 (0.689–1.533)	0.892	1.136 (0.754–1.713)	0.542
All-cause death	12 (1.7)	10 (3.8)	0.062	0.458 (0.198–1.061)	0.068	1.005 (0.384–2.629)	0.991
Cardiac death	7 (1.0)	6 (2.3)	0.136	0.446 (0.150–1.327)	0.147	0.968 (0.285–3.288)	0.958
Non-cardiac death	5 (0.7)	4 (1.5)	0.258	0.476 (0.128–1.774)	0.269	1.026 (0.174–6.046)	0.978
Recurrent MI	14 (2.0)	5 (1.9)	0.892	1.073 (0.687–2.980)	0.892	1.347 (0.471–3.856)	0.579
Any repeat revascularization	74 (10.8)	25 (9.6)	0.614	1.124 (0.714–1.768)	0.614	1.136 (0.716–1.802)	0.589
Stroke	6 (0.9)	8 (3.1)	0.013	0.293 (0.098–0.815)	0.019	2.923 (0.949–9.002)	0.062
ST (definite or probable)	1 (0.1)	1 (0.4)	0.480	3.383 (0.024–6.117)	0.497	1.140 (0.030–43.82)	0.944

MACCE, major adverse cardiac and cerebrovascular events; ST, stent thrombosis; HR, hazard ratio; CI, confidence interval; LVEF, left ventricular ejection fraction; BMI, body mass index; SBP, systolic blood pressure; DBP, diastolic blood pressure; DM, diabetes mellitus; PCI, percutaneous coronary intervention; HF, heart failure; CK-MB, creatine kinase myocardial band; eGFR, estimated glomerular filtration rate; HDL, high-density lipoprotein; LDL, low-density lipoprotein; GRACE, Global Registry of Acute Coronary Events; ACEIs, angiotensin-converting enzyme inhibitors; ARBs, angiotensin receptor blockers. ^a^ Adjusted by male sex, LVEF, BMI, SBP, DBP, symptom-to-door time, Killip class 3, hypertension, DM, dyslipidemia, previous PCI, previous HF, previous stroke, current smoker, peak CK-MB, peak troponin-I, serum creatinine, eGFR < 60 mL/min/1.73 m^2^, HDL-cholesterol, LDL-cholesterol, GRACE risk score > 140, clopidogrel, ticagrelor, prasugrel, ACEI or ARB, statin.

## Data Availability

Data are contained within the article or supplementary material.

## Supplementary Materials

The following supporting information can be downloaded at: https://www.mdpi.com/article/10.3390/jcm11164780/s1. Table S1: Results of collinearity test for MACCE, Table S2: Comparison of baseline characteristics before and after PSM, Table S3: Baseline characteristics of the total study population. Table S4: Clinical outcomes between the age ≥ 75 years and <75 years groups at 2 years. Table S5: Clinical outcomes between the age ≥ 80 years and <80 years groups at 2 years.
