# Supplementary material for: Comparison of 3-Year Outcomes between Early and Delayed Invasive Strategies in Older and Younger Adults with Non-ST-Segment Elevation Myocardial Infarction Undergoing New-Generation Drug-Eluting Stent Implantation
Source: J Clin Med. 2022 Aug 16;11(16):4780. doi: 10.3390/jcm11164780 (PMC9410439; doi:10.3390/jcm11164780)
Supplement: Supplementary file 1 [file jcm-11-04780-s001.zip › jcm-1808138-supplementary.pdf]

Supplementary

Comparison of 3-year outcomes between early and delayed invasive strategies in older and younger adults with non-ST-segment elevation myocardial infarction undergoing new-generation drug-eluting stent implantation

Running title: Early vs. delayed strategy in elderly or younger NSTEMI

Yong Hoon Kim[1,*,†], Ae-Young Her[1, †], Seung-Woon Rha[2,*], Cheol Ung Choi[2], Byoung Geol Choi[3], Ji Bak Kim[2], Soohyung Park2, Dong Oh Kang2, Ji Young Park[4], Sang-Ho Park[5] and Myung Ho Jeong[6]

Supplementary Online Contents

Supplementary material Table S1. Results of collinearity test for MACCE

Supplementary material Table S2. Comparison of baseline characteristics before and after PSM

Supplementary material Table S3. Baseline characteristics of the total study population

Supplementary material Table S4. Clinical outcomes between the age ≥75 years and <75 years groups at 2 years

Supplementary material Table S5. Clinical outcomes between the age ≥80 years and <80 years groups at 2 years

Table S1. Results of collinearity test for MACCE

	Variance Inflation Factors	Tolerance	Condition Index
Male	1.312	0.762	1.000
LVEF	1.238	0.807	3.083
BMI	1.150	0.869	3.345
SBP	2.952	0.339	3.831
DBP	2.719	0.368	4.174
Symptom-to-door time	1.025	0.976	4.191
Killip class 3	1.575	0.635	4.259
Hypertension	1.219	0.820	4.317
Diabetes mellitus	1.184	0.845	4.382
Dyslipidemia	1.041	0.961	4.428
Previous MI	5.025	0.199	4.468
Previous PCI	4.992	0.200	4.537
Previous HF	1.038	0.964	4.630
Previous stroke	1.047	0.955	4.772
Current smoker	1.283	0.780	4.998
Peak CK-MB	1.147	0.872	5.133
Peak troponin-I	1.116	0.896	5.243
Serum creatinine	1.282	0.780	5.276
eGFR <60mL/min/1.73m^2	1.578	0.634	5.586
HDL-cholesterol	1.106	0.904	5.777
LDL-cholesterol	1.156	0.865	6.008
GRACE risk score, > 140	2.610	0.383	6.729
Clopidogrel	2.918	0.919	7.010
Ticagrelor	1.088	0.921	7.334
Prasugrel	1.086	0.971	7.480
ACEI or ARB	1.030	0.955	8.940
Statin	1.047	0.949	9.643
Anitocagulant	1.054	0.905	11.074
Left main (IRA)	1.105	0.882	13.595
Multivessel disease	1.134	0.879	16.132
Pre-PCI TIMI flow grade 0/1	1.137	0.934	18.398
GP IIb/IIIa inhibitor	1.070	0.890	18.788
Transradial approach	1.124	0.927	21.602
IVUS/OCT	1.078	0.353	23.476
FFR	1.019	0.982	25.946
Other stents	1.013	0.987	30.859
Stent diameter	1.130	0.885	35.889
Stent length	2.492	0.401	50.954
Number of stents	2.433	0.411	86.922

MACE, major adverse cardiac events; LVEF, left ventricular ejection fraction; BMI, body mass index; SBP, systolic blood pressure; DBP, diastolic blood pressure; CPR, cardiopulmonary resuscitation; PCI, percutaneous coronary intervention; CABG, coronary artery bypass graft; HF, heart failure; CVA, cerebrovascular accidents; CK-MB, creatine

kinase myocardial band; NT-ProBNP, N-terminal pro-brain natriuretic peptide; eGFR, estimated glomerular filtration rate; LDL, low density lipoprotein; ACEI, angiotensin converting enzyme inhibitor; ARB, angiotensin receptor blocker; BB, beta-blocker; CCB, calcium channel blocker; IRA, infarct-related artery; LAD, left anterior descending coronary artery; LCx, left circumflex coronary artery; RCA, right coronary artery; ACC/AHA, American College of Cardiology/American Heart Association; TIMI, thrombolysis in myocardial infarction; GP, glycoprotein; IVUS, intravascular ultrasound;, OCT, optical coherence tomography; FFR, fractional flow reserve; ZES, zotarolimus-eluting stent; EES, everolimus-eluting stent; BES, biolimus-eluting stent.

Table S2. Comparison of baseline characteristics before and after PSM

Variables	Entire patients (n = 4,513)				PSM patients (n = 2,318)			
	Group A1+B1 Early invasive (n = 3300)	Group A2+B2 Delayed invasive (n = 1213)	*p* value	SD	Early invasive (n = 1159)	Delayed invasive (n = 1159)	*p* value	SD
Male, n (%)	2403 (72.8)	884 (72.9)	0.968	-0.22	840 (72.4)	836 (72.1)	0.892	0.07
LVEF, %	54.6 ± 10.1	53.3 ± 11.8	0.001	1.20	53.8 ± 9.9	53.7 ± 11.4	0.633	0.09
BMI, kg/m^2	24.1 ± 3.3	24.1 ± 3.3	0.832	0.07	24.2 ± 3.2	24.1 ± 3.2	0.637	0.19
SBP, mmHg	134.9 ± 25.7	138.5 ± 26.6	<0.001	-1.38	138.8 ± 26.8	138.0 ± 26.4	0.457	0.30
DBP, mmHg	81.3 ± 15.3	81.8 ± 15.2	0.331	-0.33	82.1 ± 15.5	81.8 ± 15.1	0.593	0.19
Symptom-to-door time, h	6.9 (2.5-24.0)	6.2 (2.1-27.7)	0.019	-0.92	7.0 (2.4-24.4)	6.2 (2.1-27.3)	0.847	-0.22
Killip class 3	246 (7.5)	132 (10.9)	<0.001	-1.18	104 (9.0)	122 (10.5)	0.357	-0.51
Hypertension, n (%)	1712 (51.9)	670 (55.2)	0.045	-0.66	634 (54.7)	636 (54.9)	0.933	-0.04
Diabetes mellitus, n (%)	975 (29.5)	381 (31.4)	0.226	-0.42	338 (29.2)	357 (30.8)	0.415	-0.35
Dyslipidemia, n (%)	379 (11.5)	175 (14.4)	0.009	-0.87	152 (13.1)	165 (14.2)	0.468	-0.32
Previous MI, n (%)	209 (6.3)	86 (7.1)	0.377	-0.32	76 (6.6)	80 (6.9)	0.804	-0.12
Previous PCI, n (%)	178 (5.4)	67 (5.5)	0.882	-0.04	59 (5.1)	63 (5.4)	0.780	-0.14
Previous CABG, n (%)	8 (0.2)	4 (0.3)	0.774	-0.20	6 (0.5)	3 (0.3)	0.507	0.31
Previous HF, n (%)	36 (1.1)	21 (1.7)	0.098	-0.51	20 (1.7)	21 (1.8)	0.875	-0.07
Previous stroke, n (%)	184 (5.6)	80 (6.6)	0.198	-0.42	75 (6.5)	71 (6.1)	0.789	0.16
Current smokers, n (%)	1245 (37.7)	411 (33.9)	0.018	0.79	403 (34.8)	397 (34.3)	0.827	0.10
Peak CK-MB, mg/dL	24.6 (6.7-90.2)	14.4 (4.8-49.3)	<0.001	2.57	15.4 (4.7-50.7)	15.0 (4.8-52.9)	0.847	-0.08
Peak Troponin-I, ng/mL	11.9 (2.4-23.0)	4.9 (1.0-20.6)	<0.001	2.40	6.1 (1.3-21.6)	5.0 (1.2-21.6)	0.874	-0.02
Blood glucose, mg/dL	156.1 ± 73.1	160.6 ± 79.9	0.082	-0.58	157.0 ± 74.4	159.3 ± 77.1	0.465	-0.30
Hs-CRP (mg/dL)	1.30 ± 2.90	1.46 ± 5.80	0.335	-0.35	1.39 ± 3.90	1.32 ± 2.51	0.600	0.21
Serum creatinine (mg/L)	1.08 ± 1.21	1.23 ± 1.54	0.002	-1.08	1.15 ± 1.34	1.20 ± 1.48	0.453	-0.35
eGFR <60mL/min/1.73m^2, n (%)	763 (23.1)	355 (29.3)	<0.001	-1.41	286 (24.7)	314 (27.1)	0.308	-0.55

Total cholesterol, mg/dL	180.4 ± 44.0	178.1 ± 43.5	0.116	0.52	180.0 ± 43.5	178.6 ± 43.5	0.440	0.32
Triglyceride, mg/L	132.7 ± 84.5	133.3 ± 89.3	0.862	-0.10	137.5 ± 97.9	134.1 ± 95.7	0.507	0.35
HDL cholesterol, mg/L	42.6 ± 11.1	43.4 ± 11.4	0.038	-0.71	43.1 ± 10.9	43.3 ± 11.3	0.704	-0.18
LDL cholesterol, mg/L	114.6 ± 36.2	111.1 ± 35.7	0.004	0.97	112.4 ± 35.2	111.9 ± 35.7	0.734	0.14
GRACE risk score	128.0 ± 38.8	131.8 ± 42.1	0.006	-0.94	129.3 ± 38.5	129.5 ± 41.6	0.955	-0.05
> 140, n (%)	1150 (34.8)	471 (38.8)	0.013	-0.83	432 (37.3)	424 (36.6)	0.795	0.15
Atrial fibrillation, n (%)	119 (3.6)	57 (4.7)	0.099	-0.55	62 (5.3)	54 (4.7)	0.505	0.27
ST-depression, n (%)	726 (22.0)	260 (21.4)	0.715	0.15	247 (21.3)	255 (22.0)	0.724	-0.17
T-wave inversion, n (%)	661 (20.0)	274 (22.6)	0.062	-0.64	282 (24.3)	257 (22.2)	0.238	0.45
Discharge medications, n (%)								
Aspirin, n (%)	3278 (99.3)	1203 (99.2)	0.576	0.12	1150 (99.2)	1149 (99.1)	0.818	0.10
Clopidogrel, n (%)	2316 (70.2)	946 (78.0)	<0.001	-1.79	882 (76.1)	896 (77.3)	0.523	-0.28
Ticagrelor, n (%)	644 (19.5)	186 (15.3)	0.001	1.11	200 (17.3)	184 (15.9)	0.402	0.37
Prasugrel, n (%)	340 (10.3)	81 (6.7)	<0.001	1.29	77 (6.6)	79 (6.8)	0.934	-0.08
BBs, n (%)	2845 (86.2)	1027 (84.7)	0.187	0.43	998 (86.1)	987 (85.2)	0.621	0.25
ACEI or ARBs, n (%)	2784 (84.4)	968 (79.8)	<0.001	1.20	946 (81.6)	939 (81.0)	0.709	0.15
Statin, n (%)	3165 (95.9)	1142 (94.1)	0.012	0.83	1134 (96.1)	1110 (95.8)	0.803	0.15
Anticoagulant, n (%)	61 (1.8)	35 (2.9)	0.036	-0.73	20 (1.7)	24 (2.1)	0.593	-0.29
Infarct-related artery								
Left main, n (%)	83 (2.5)	48 (4.0)	0.012	-0.85	37 (3.2)	45 (3.9)	0.431	-0.37
LAD, n (%)	1407 (42.6)	524 (43.2)	0.735	0.12	496 (42.8)	497 (42.9)	0.967	-0.02
LCx, n (%)	859 (26.0)	291 (24.0)	0.166	0.46	283 (24.4)	280 (24.2)	0.884	0.05
RCA, n (%)	951 (28.8)	350 (28.9)	0.981	-0.02	343 (29.6)	337 (29.1)	0.820	0.11
Multivessel disease, n (%)	1782 (54.0)	723 (59.6)	0.001	-1.13	676 (58.3)	682 (58.8)	0.800	0.05
ACC/AHA type B2/C lesions	2786 (84.4)	1011 (83.3)	0.380	0.30	996 (85.9)	978 (84.4)	0.397	-0.10
Pre-PCI TIMI flow grade 0/1	1393 (42.2)	376 (31.0)	<0.001	2.34	364 (31.4)	369 (31.8)	0.858	-0.09
GP IIb/IIIa inhibitor	307 (9.3)	84 (6.9)	0.012	0.88	87 (7.5)	84 (7.2)	0.812	-0.11
Transradial approach	1740 (52.7)	601 (49.5)	0.060	0.64	585 (50.5)	581 (50.1)	0.868	0.08
IVUS/OCT, n (%)	767 (23.2)	376 (31.0)	<0.001	-1.76	321 (27.7)	340 (29.3)	0.382	-0.35
FFR, n (%)	60 (1.8)	47 (3.9)	<0.001	-1.27	38 (3.3)	34 (2.9)	0.720	0.23

Drug-eluting stents[a]								
ZES, n (%)	793 (24.0)	297 (24.5)	0.752	-0.12	299 (25.8)	288 (24.8)	0.633	0.23
EES, n (%)	1,738 (52.7)	626 (51.6)	0.528	0.22	591 (51.0)	601 (51.9)	0.678	-0.18
BES, n (%)	666 (20.2)	269 (22.2)	0.143	-0.49	252 (21.7)	249 (21.5)	0.920	0.05
Others, n (%)	103 (3.1)	21 (1.7)	0.010	0.92	17 (1.5)	21 (1.8)	0.624	-0.24
Stent diameter (mm)	3.08 ± 0.42	3.06 ± 0.43	0.114	0.47	3.08 ± 0.42	3.06 ± 0.42	0.298	0.47
Stent length (mm)	29.4 ± 13.8	30.5 ± 14.8	0.023	-0.77	31.1 ± 14.9	30.4 ± 14.6	0.332	0.47
Number of stents	1.19 ± 0.44	1.24 ± 0.49	0.002	-1.07	1.25 ± 0.49	1.23 ± 0.48	0.521	0.41

Values are means ± standard deviation or median (interquartile range) or numbers and percentages. The *p* values for continuous data were obtained from the unpaired t-test. The *p* values for categorical data from chi-square or Fisher's exact test.

PSM propensity-score matched, SD standardized mean difference, BMI, body mass index; SBP, systolic blood pressure; DBP, diastolic blood pressure; MI, myocardial infarction; PCI, percutaneous coronary intervention; CABG, coronary artery bypass graft; HF, heart failure; CK-MB, creatine kinase myocardial band; Hs-CRP, high sensitivity C-reactive protein; eGFR, estimated glomerular filtration rate; HDL, high-density lipoprotein; LDL, low-density lipoprotein; GRACE, Global Registry of Acute Coronary Events; BBs, ß-blockers; ACEIs, angiotensin-converting enzyme inhibitors; ARBs, angiotensin receptor blockers; LAD, left anterior descending artery; LCx, left circumflex artery; RCA, right coronary artery; ACC/AHA, American College of Cardiology/American Heart Association; TIMI, thrombolysis in myocardial infarction; GP, glycoprotein; IVUS, intravascular ultrasound; OCT, optical coherence tomography; FFR, fractional flow reserve; ZES, zotarolimus-eluting stent; EES, everolimus-eluting stent; BES, biolimus-eluting stent. [a]Drug-eluting stents were composed of ZES (Resolute Integrity stent; Medtronic, Inc., Minneapolis, MN), EES (Xience Prime stent, Abbott Vascular, Santa Clara, CA; or Promus Element stent, Boston Scientific, Natick, MA), and BES (BioMatrix Flex stent, Biosensors International, Morges, Switzerland; or Nobori stent, Terumo Corporation, Tokyo, Japan).

Table S3. Baseline characteristics of the total study population.

Variables	Early invasive (n = 3,300)			Delayed invasive (n = 1,213)		
	Group A1 Age, ≥65 years (n = 1,612)	Group B1 Age, <65 years (n = 1,688)	*p* value	Group A2 Age, ≥65 years (n = 641)	Group B2 Age, <65 years (n = 572)	*p* value
Male, n (%)	927 (57.5)	1476 (87.4)	<0.001	371 (57.9)	513 (89.7)	<0.001
Age, years	74.3 ± 5.8	54.4 ± 7.3	<0.001	75.0 ± 5.9	54.5 ± 7.2	<0.001
LVEF, %	53.2 ± 10.6	55.9 ± 9.4	<0.001	51.6 ± 12.3	55.1 ± 10.9	<0.001
BMI, kg/m^2	23.2 ± 3.1	25.0 ± 3.2	<0.001	23.5 ± 3.3	24.8 ± 3.1	<0.001
SBP, mmHg	133.5 ± 26.4	137.0 ± 25.8	<0.001	135.4 ± 25.8	139.2 ± 25.8	0.398
DBP, mmHg	80.4 ± 15.7	83.9 ± 15.8	<0.001	81.3 ± 14.8	83.8 ± 15.1	<0.001
Symptom-to-door time, h	8.0 (3.0-28.4)	5.8 (2.0-19.3)	<0.001	8.8 (3.0-45.3)	4.5 (1.6-23.9)	<0.001
Door-to-balloon time, h	6.0 (2.9-16.1)	6.9 (3.0-16.1)	0.529	46.4 (31.8-71.6)	43.2 (29.8-58.6)	0.244
Killip class ≥ 3	181 (11.2)	65 (3.9)	<0.001	98 (15.3)	34 (5.9)	<0.001
Hypertension, n (%)	1050 (65.1)	662 (39.2)	<0.001	427 (66.6)	243 (42.5)	<0.001
Diabetes mellitus, n (%)	567 (35.2)	408 (24.2)	<0.001	227 (35.4)	154 (26.9)	0.001
Dyslipidemia, n (%)	154 (9.6)	225 (13.3)	0.001	83 (12.9)	92 (16.1)	0.121
Previous MI, n (%)	136 (8.4)	73 (4.3)	<0.001	48 (7.5)	388 (6.6)	0.578
Previous PCI, n (%)	112 (6.9)	66 (3.9)	<0.001	33 (5.1)	34 (5.9)	0.545
Previous CABG, n (%)	6 (0.4)	2 (0.1)	0.170	3 (0.5)	1 (0.2)	0.627
Previous HF, n (%)	27 (1.7)	9 (0.5)	0.002	15 (2.3)	6 (1.0)	0.121
Previous stroke, n (%)	124 (7.7)	60 (3.6)	<0.001	57 (8.9)	23 (4.0)	0.001
Current smokers, n (%)	324 (20.1)	921 (54.6)	<0.001	102 (15.9)	309 (54.0)	<0.001
Peak CK-MB, mg/dL	20.9 (6.4-78.6)	29.0 (7.2-99.0)	0.228	13.9 (5.0-42.6)	15.6 (4.6-56.7)	0.640
Peak Troponin-I, ng/mL	10.6 (2.1-22.1)	14.3 (2.8-23.1)	0.146	46.7 (11.1-188.9)	53.7 (10.3-211.3)	0.661
Blood glucose, mg/dL	158.6 ± 72.7	153.6 ± 73.4	0.049	162.1 ± 80.2	158.9 ± 79.6	0.491
Hs-sensitivity CRP (mg/dL)	1.53 ± 3.24	1.07 ± 2.50	<0.001	1.78 ± 7.72	1.11 ± 2.10	0.035
Serum creatinine (mg/L)	1.12 ± 1.15	1.04 ± 1.27	0.050	1.26 ± 1.34	1.21 ± 1.73	0.548
eGFR <60mL/min/1.73m^2, n (%)	570 (35.4)	193 (11.4)	<0.001	269 (42.0)	86 (15.0)	<0.001

Total cholesterol, mg/dL	171.9 ± 43.3	188.5 ± 43.1	<0.001	171.7 ± 44.1	185.3 ± 41.9	<0.001
Triglyceride, mg/L	111.7 ± 71.8	152.7 ± 96.3	<0.001	112.8 ± 82.7	156.2 ± 94.3	<0.001
HDL cholesterol, mg/L	43.1 ± 11.4	42.1 ± 10.8	0.010	44.5 ± 82.7	42.2 ± 10.6	<0.001
LDL cholesterol, mg/L	108.7 ± 34.7	120.2 ± 36.8	<0.001	106.0 ± 35.3	116.9 ± 35.3	<0.001
GRACE risk score	151.2 ± 34.5	105.8 ± 28.4	<0.001	154.4 ± 36.7	106.5 ± 32.3	<0.001
> 140, n (%)	979 (60.7)	171 (10.1)	<0.001	390 (60.8)	81 (14.2)	<0.001
Atrial fibrillation, n (%)	93 (5.8)	26 (1.5)	<0.001	44 (6.9)	13 (2.3)	<0.001
ST-depression, n (%)	392 (24.3)	334 (19.8)	0.002	157 (24.5)	103 (18.0)	0.006
T-wave inversion, n (%)	370 (23.0)	291 (17.2)	<0.001	155 (24.2)	119 (20.8)	0.169
Discharge medications, n (%)						
Aspirin, n (%)	1600 (99.3)	1678 (99.4)	0.592	635 (99.1)	568 (99.3)	0.649
Clopidogrel, n (%)	1251 (77.6)	1065 (63.1)	<0.001	540 (84.2)	406 (71.0)	<0.001
Ticagrelor, n (%)	283 (17.6)	361 (21.4)	0.006	77 (12.0)	109 (19.1)	0.001
Prasugrel, n (%)	78 (4.8)	262 (15.5)	<0.001	24 (3.7)	57 (10.0)	0.001
BBs, n (%)	1354 (84.0)	1491 (88.3)	<0.001	542 (84.6)	485 (84.8)	0.910
ACEI or ARBs, n (%)	1361 (84.4)	1423 (84.3)	0.924	506 (78.9)	462 (80.8)	0.428
Statin, n (%)	1534 (95.2)	1631 (96.6)	0.034	601 (93.8)	541 (94.6)	0.543
Anticoagulant, n (%)	50 (3.1)	11 (0.7)	<0.001	25 (3.9)	10 (1.7)	0.026
Infarct-related artery						
Left main, n (%)	50 (3.1)	33 (2.0)	0.045	25 (3.9)	23 (4.0)	0.914
LAD, n (%)	684 (42.4)	723 (42.8)	0.833	286 (44.6)	238 (41.6)	0.291
LCx, n (%)	400 (24.8)	459 (27.2)	0.122	141 (22.0)	150 (26.2)	0.085
RCA, n (%)	478 (29.7)	473 (28.0)	0.301	189 (29.5)	161 (28.1)	0.608
Multivessel disease, n (%)	971 (60.2)	811 (48.0)	<0.001	423 (66.0)	300 (52.4)	<0.001
ACC/AHA type B2/C lesions	1373 (85.2)	1413 (83.7)	0.246	544 (84.9)	467 (81.6)	0.143
Pre-PCI TIMI flow grade 0/1	633 (39.3)	760 (45.0)	0.001	199 (31.0)	177 (30.9)	0.970
GP IIb/IIIa inhibitor	133 (8.3)	174 (10.3)	0.048	43 (6.7)	41 (7.2)	0.821
Transradial approach	781 (48.4)	959 (56.8)	<0.001	309 (48.2)	292 (51.0)	0.323
IVUS/OCT, n (%)	346 (21.5)	421 (24.9)	0.019	174 (27.1)	202 (35.3)	0.002
FFR, n (%)	27 (1.7)	33 (2.0)	0.603	23 (3.6)	24 (4.2)	0.656

Drug-eluting stents[a]						
ZES, n (%)	374 (23.2)	419 (24.8)	0.276	155 (24.2)	142 (24.8)	0.795
EES, n (%)	860 (53.3)	878 (52.0)	0.442	332 (51.8)	294 (51.4)	0.891
BES, n (%)	326 (20.2)	340 (20.1)	0.954	144 (22.5)	125 (21.9)	0.798
Others, n (%)	52 (3.2)	51 (3.0)	0.764	10 (1.6)	11 (1.9)	0.665
Stent diameter (mm)	3.04 ± 0.40	3.12 ± 0.43	<0.001	3.03± 0.41	3.10± 0.44	0.008
Stent length (mm)	30.2 ± 14.4	28.6 ± 13.2	0.001	31.1 ± 14.9	29.8 ± 14.5	0.129
Number of stents	1.22 ± 0.46	1.17 ± 0.42	0.002	1.26 ± 0.50	1.22 ± 0.47	0.105

Values are means ± standard deviation or median (interquartile range) or numbers and percentages. The *p* values for continuous data were obtained from the unpaired t-test. The *p* values for categorical data from chi-square or Fisher's exact test.

LVEF, left ventricular ejection fraction; BMI, body mass index; SBP, systolic blood pressure; DBP, diastolic blood pressure; MI, myocardial infarction; PCI, percutaneous coronary intervention; CABG, coronary artery bypass graft; HF, heart failure; CK-MB, creatine kinase myocardial band; Hs-CRP, high sensitivity C-reactive protein; eGFR, estimated glomerular filtration rate; HDL, high-density lipoprotein; LDL, low-density lipoprotein; GRACE, Global Registry of Acute Coronary Events; BBs, ß-blockers; ACEIs, angiotensin-converting enzyme inhibitors; ARBs, angiotensin receptor blockers; LAD, left anterior descending artery; LCx, left circumflex artery; RCA, right coronary artery; ACC/AHA, American College of Cardiology/American Heart Association; TIMI, thrombolysis in myocardial infarction; GP, glycoprotein; IVUS, intravascular ultrasound; OCT, optical coherence tomography; FFR, fractional flow reserve; ZES, zotarolimus-eluting stent; EES, everolimus-eluting stent; BES, biolimus-eluting stent. [a]Drug-eluting stents were composed of ZES (Resolute Integrity stent; Medtronic, Inc., Minneapolis, MN), EES (Xience Prime stent, Abbott Vascular, Santa Clara, CA; or Promus Element stent, Boston Scientific, Natick, MA), and BES (BioMatrix Flex stent, Biosensors International, Morges, Switzerland; or Nobori stent, Terumo Corporation, Tokyo, Japan.

Table S4. Clinical outcomes between the age ≥75 years and <75 years groups at 2 years

Outcomes	Group A (Age, ≥75 years, *n* = 984)		Log-rank	Unadjusted		Multivariable-Adjusted[a]	
	Group A1 Early invasive (*n* = 684)	Group A2 Delayed invasive (*n* = 300)		HR (95% CI)	*p*	HR (95% CI)	*p*
MACCE	125 (18.3)	47 (15.7)	0.340	1.177 (0.842-1.646)	0.340	1.218 (0.867-1.710)	0.255
All-cause death	74 (10.8)	25 (8.3)	0.229	1.320 (0.838-2.076)	0.231	1.424 (0.897-2.260)	0.133
Cardiac death	45 (6.6)	15 (5.0)	0.330	1.337 (0.746-2.399)	0.329	1.548 (0.852-2.814)	0.152
Non-cardiac death	29 (4.2)	10 (3.3)	0.484	1.283 (0.630-2.652)	0.484	1.330 (0.638-2.774)	0.446
Recurrent MI	29 (4.2)	12 (4.1)	0.835	1.074 (0.548-2.105)	0.835	1.103 (0.558-2.179)	0.778
Any repeat revascularization	52 (8.0)	21 (7.2)	0.714	1.099 (0.662-1.825)	0.715	1.128 (0.676-1.882)	0.645
Stroke	16 (2.4)	13 (4.5)	0.100	0.546 (0.263-1.135)	0.105	1.842 (0.880-3.856)	0.214
ST (definite or probable)	4 (0.6)	2 (0.7)	0.888	0.886 (0.162-4.836)	0.869	1.125 (0.189-6.701)	0.897
Outcomes	Group B (Age, <75 years, *n* = 3,529)		Log-rank	Unadjusted		Multivariable-Adjusted[a]	
	Group B1 Early invasive (*n* = 2,816)	Group B2 Delayed invasive (*n* = 913)		HR (95% CI)	*p*	HR (95% CI)	*p*
MACCE	325 (12.4)	106 (11.6)	0.515	1.015 (0.864-1.339)	0.515	1.144 (0.918-1.427)	0.232
All-cause death	68 (2.6)	36 (3.9)	0.038	0.654 (0.436-0.979)	0.038	1.148 (0.753-1.750)	0.521
Cardiac death	31 (1.2)	22 (2.4)	0.009	0.488 (0.283-0.843)	0.010	1.463 (0.827-2.588)	0.191
Non-cardiac death	37 (1.4)	14 (1.5)	0.773	0.913 (0.494-1.689)	0.773	1.154 (0.610-2.183)	0.660
Recurrent MI	73 (2.8)	25 (2.8)	0.955	1.013 (0.643-1.596)	0.955	1.077 (0.682-1.703)	0.750
Any repeat revascularization	249 (9.6)	72 (8.0)	0.150	1.212 (0.932-1.576)	0.151	1.245 (0.957-1.620)	0.102
Stroke	45 (1.7)	19 (2.1)	0.461	0.818 (0.478-1.398)	0.462	1.133 (0.659-1.948)	0.651
ST (definite or probable)	14 (0.5)	5 (0.6)	0.955	0.971 (0.350-2.696)	0.955	1.099 (0.391-3.086)	0.858

MACCE, major adverse cardiac and cerebrovascular events; ST, stent thrombosis; HR, hazard ratio; CI, confidence interval; LVEF, left ventricular ejection fraction; BMI, body mass index; SBP, systolic blood pressure; DBP, diastolic blood pressure; DM, diabetes mellitus; PCI, percutaneous coronary intervention; HF, heart failure; CK-MB, creatine kinase myocardial band; eGFR, estimated glomerular filtration rate; HDL, high-density lipoprotein; LDL, low-density lipoprotein; GRACE, Global Registry of Acute Coronary Events; ACEIs, angiotensin-converting enzyme inhibitors; ARBs, angiotensin receptor blockers.

[a]Adjusted by male sex, LVEF, BMI, SBP, DBP, symptom-to-door time, Killip class 3, hypertension, DM, dyslipidemia, previous PCI,

previous HF, previous stroke, current smoker, peak CK-MB, peak troponin-I, serum creatinine, eGFR <60mL/min/1.73m^2, HDL-cholesterol, LDL-cholesterol, GRACE risk score >140, clopidogrel, ticagrelor, prasugrel, ACEI or ARB, and statin

Table S5. Clinical outcomes between the age ≥80 years and <80 years groups at 2 years

Outcomes	Group A (Age, ≥80 years, *n* = 410)		Log-rank	Unadjusted		Multivariable-Adjusted[a]	
	Group A1 Early invasive (*n* = 280)	Group A2 Delayed invasive (*n* = 130)		HR (95% CI)	*p*	HR (95% CI)	*p*
MACCE	61 (21.8)	21 (16.2)	0.180	1.402 (0.884-2.302)	0.182	1.438 (0.872-2.371)	0.154
All-cause death	41 (14.6)	12 (9.2)	0.126	1.643 (0.863-3.126)	0.130	1.683 (0.880-3.221)	0.116
Cardiac death	27 (9.9)	7 (5.4)	0.143	1.856 (0.808-4.261)	0.145	1.913 (0.824-4.442)	0.131
Non-cardiac death	14 (4.7)	5 (3.8)	0.568	1.345 (0.484-3.734)	0.570	1.615 (0.534-4.886)	0.396
Recurrent MI	15 (5.6)	3 (2.3)	0.160	2.423 (0.702-8.371)	0.162	3.057 (0.835-11.19)	0.091
Any repeat revascularization	20 (7.7)	8 (6.5)	0.663	1.200 (0.528-2.724)	0.663	1.226 (0.537-2.799)	0.629
Stroke	7 (2.6)	4 (3.3)	0.769	0.832 (0.243-2.842)	0.769	1.120 (0.321-3.906)	0.859
ST (definite or probable)	1 (0.4)	-	-	-	-	-	-
Outcomes	Group B (Age, <80 years, *n* = 4,103)		Log-rank	Unadjusted		Multivariable-Adjusted[a]	
	Group B1 Early invasive (*n* = 3,020)	Group B2 Delayed invasive (*n* = 1,083)		HR (95% CI)	*p*	HR (95% CI)	*p*
MACCE	389 (12.9)	132 (12.2)	0.573	1.058 (0.869-1.289)	0.573	1.137 (0.929-1.383)	0.217
All-cause death	101 (3.3)	49 (4.5)	0.074	0.734 (0.522-1.032)	0.075	1.014 (0.711-1.446)	0.939
Cardiac death	49 (1.6)	30 (2.7)	0.018	0.582 (0.369-0.916)	0.019	1.233 (0.768-1.979)	0.386
Non-cardiac death	52 (1.7)	19 (1.8)	0.921	0.974 (0.576-1.647)	0.921	1.253 (0.727-2.160)	0.416
Recurrent MI	87 (2.9)	34 (3.2)	0.646	0.911 (0.613-1.354)	0.645	1.034 (0.694-1.540)	0.871
Any repeat revascularization	281 (9.5)	85 (8.0)	0.163	1.188 (0.932-1.515)	0.163	1.222 (0.958-1.559)	0.106
Stroke	54 (1.8)	28 (2.6)	0.102	0.685 (0.434-1.081)	0.104	1.359 (0.857-2.154)	0.192
ST (definite or probable)	17 (0.6)	7 (0.7)	0.748	0.866 (0.359-2.088)	0.748	1.017 (0.417-2.481)	0.970

MACCE, major adverse cardiac and cerebrovascular events; ST, stent thrombosis; HR, hazard ratio; CI, confidence interval; LVEF, left ventricular ejection fraction; BMI, body mass index; SBP, systolic blood pressure; DBP, diastolic blood pressure; DM, diabetes mellitus; PCI, percutaneous coronary intervention; HF, heart failure; CK-MB, creatine kinase myocardial band; eGFR, estimated glomerular filtration rate; HDL, high-density lipoprotein; LDL, low-density lipoprotein; GRACE, Global Registry of Acute Coronary Events; ACEIs, angiotensin-converting enzyme inhibitors; ARBs, angiotensin receptor blockers.

[a]Adjusted by male sex, LVEF, BMI, SBP, DBP, symptom-to-door time, Killip class 3, hypertension, DM, dyslipidemia, previous PCI,

previous HF, previous stroke, current smoker, peak CK-MB, peak troponin-I, serum creatinine, eGFR <60mL/min/1.73m^2, HDL-cholesterol, LDL-cholesterol, GRACE risk score >140, clopidogrel, ticagrelor, prasugrel, ACEI or ARB, and statin
